# Fascicle length does increase in response to longitudinal resistance training and in a contraction-mode specific manner

**DOI:** 10.1186/s40064-015-1548-8

**Published:** 2016-01-28

**Authors:** Martino V. Franchi, Philip J. Atherton, Constantinos N. Maganaris, Marco V. Narici

**Affiliations:** MRC-ARUK Centre of Excellence for Musculoskeletal Ageing Research, School of Medicine, University of Nottingham, Derby, DE22 3DT UK; Research Institute for Sport and Exercise Sciences, Liverpool John Moores University, Liverpool, UK

Dear Editor:

Morphological adaptations of skeletal muscle to resistance exercise training (RET) have been the subject of many studies: essentially, muscle hypertrophy is achieved by a structural remodelling of the contractile machinery, which can be assessed macroscopically by investigating changes in muscle architecture (i.e. fascicle length, Lf; pennation angle, PA; muscle thickness, MT) (Gans 1982; Narici 1999; Lieber and Fridén 2000, 2001; Reeves et al. 2004, 2005). A thorough understanding of muscle architecture is indeed fundamental when interpreting training-induced changes in muscle function given its key role as determinant of muscle mechanical properties (Narici et al. 2015; Lieber and Fridén 2000).

In a recent study by Fukutani and Kurihara (2015) published in *SpringerPlus* (2015, 4:341), the authors investigated differences in Lf between resistance trained and untrained individuals using a cross-sectional design: the main conclusion being made was that Lf was not associated with muscle hypertrophy on the basis that no significant differences in Lf were found between the groups. The authors claimed that fascicle length does not increase with resistance training.

Some fundamental considerations arise from these findings. Skeletal muscle hypertrophy in response to RET is mainly accomplished with the addition of new contractile material as a result of enhanced muscle myofibrillar protein synthesis after exercise (Glass 2003; Atherton and Smith 2012). Moreover, it is well established that the longitudinal post-natal growth of mammal muscle is associated with the increased in length and size of muscle fibres (Goldspink 1968; Williams and Goldspink 1971; Russell et al. 2000). Seminal pre-clinical studies previously showed that skeletal muscle responds to passive and intermittent stretch by adding new sarcomeres in-series (Holly et al. 1980; Goldspink 1985; Williams et al. 1988; Williams 1990), a phenomenon that occurs also in response to exercise regimes/overload, especially when including lengthening muscle actions (Goldspink 1999; Proske and Morgan 2001). Greater addition of serial sarcomeres was found in rats after downhill compared to uphill running (Lynn and Morgan 1994; Butterfield et al. 2005), reinforcing the concept of muscle longitudinal growth being intimately related to lengthening contractions. Indeed, the addition of sarcomeres in series (and thus increased Lf) appears to be one of the main “protective” mechanisms after eccentric exercise induced muscle damage (Morgan and Talbot 2002).

Further support to these observations on animal muscle can be found in numerous studies investigating architectural responses to RET, directly in humans. Interestingly, Fukutani and Kurihara stated it as controversial as to whether Lf increases after RET: however the number of reports showing no increases in Lf in response to exercise is limited (Blazevich et al. 2007b; Erskine et al. 2010; Ema et al. 2013) compared to those that demonstrated an increase in Lf after either conventional resistance, isokinetic, isoinertial or even marathon training (Morgan and Proske 2004; Seynnes et al. 2007; Blazevich et al. 2007a; Potier et al. 2009; Reeves et al. 2009; Baroni et al. 2013; Franchi et al. 2014, 2015; Sharifnezhad et al. 2014; McMahon et al. 2014; Murach et al. 2015). But, most importantly, it was recently reported by our group that, in both young and older populations, architectural changes, such as increases in Lf, are somewhat contraction-specific (Reeves et al. 2009; Franchi et al. 2014, 2015). That is, concentric loading promotes increases in PA, reflecting preferential addition of sarcomeres in parallel, whereas eccentric training favours the increase of Lf through the addition of sarcomeres in series. It is our opinion that these investigations should have been cited in Fukutani and Kurihara’s manuscript. Furthermore, considering the substantial number of longitudinal studies that have showed significant changes in Lf and muscle architecture after RET, the adoption of such a cross-sectional study design calls into question the validity of these conclusions. Moreover, the investigation was performed on recreationally active volunteers (the untrained group, with “no experience in regular RET”) compared to a group of “resistance exercise trained” participants, either body builders or rugby players (i.e. the number of bodybuilders/rugby players was not specified). Taking into account the aforementioned considerations on the contraction-specificity of architectural responses, the individual history of resistance training in both groups should have been accounted for. Kawakami and colleagues (1993) previously reported that PA and MT are greater in bodybuilders compared to untrained and moderately trained subjects (Lf was not investigated), but Abe et al. (2000, 2001), showed that Lf is greater in elite male 100 m-sprinters compared to elite long-distance runners and to non-sprinters. Rather than being innate factors, as Fukutani and Kurihara argue, architectural adaptations such as increases in Lf are indeed detectable longitudinally and are training/contraction-specific (Blazevich et al. 2003; Franchi et al. 2014, 2015). In addition, Lf was measured as a straight line in Fukutani and Kurihara’s study: while this might not represent a problem in the untrained group, in hypertrophied muscle, instead, fascicles show a significantly greater curvature, which partially explains the increased pennation occurring with hypertrophy (clearly visible in bodybuilders muscle) (Kawakami et al. 1993). Since the fascicle curvature was neglected by the methodological approach used to measure Lf, the true Lf values could have been underestimated in the resistance-trained group. Therefore, Lf may have gone undetected as a result of the simplicity of the morphometric analyses implemented. Thus, the chances are that Fukutani and Kurihara’s results were biased by the non-longitudinal study design and by the possible underestimation of Lf due to specific methodological approach. We agree that in some cases “muscle hypertrophy is not necessarily accompanied with increase in Lf” (Fukutani and Kurihara 2015), but these cases can only be truly determined by tightly controlled longitudinal studies.

We are of the opinion that the nature of fascicle length (Lf) increase is highly dependent on which type of contraction and mechanical stimulus is predominant in specific RET programmes: thus, data on muscle architecture features should be cautiously interpreted, as crucial in the understanding of muscle structural remodelling and its functional characteristics.

## Abbreviations

RET: resistance-exercise training
Lf: fascicle length
PA: pennation angle
MT: muscle thickness
